# Effect of the Duty Cycle of Flower-like Silver Nanostructures Fabricated with a Lyotropic Liquid Crystal on the SERS Spectrum

**DOI:** 10.3390/molecules26216522

**Published:** 2021-10-28

**Authors:** Shen Zhang, Zhihui Jiang, Yijin Liang, Yili Shen, Hongmin Mao, Huijuan Sun, Xin Zhao, Xiaoping Li, Wusheng Hu, Guoding Xu, Zhaoliang Cao

**Affiliations:** 1 Jiangsu Key Laboratory of Micro and Nano Heat Fluid Flow Technology and Energy Application, School of Physical Science and Technology, Suzhou University of Science and Technology, Suzhou 215009, China; zhangshen1018@foxmail.com (S.Z.); zhihui_jiang@163.com (Z.J.); hongminmao@mail.usts.edu.cn (H.M.); guodingxu@163.com (G.X.); 2 Shanghai Institute of Satellite Engineering, China Aerospace Science and Technology Corporation, Shanghai 201109, China; laofly86128@163.com (Y.L.); shenyili007@sina.com (Y.S.); 3 Institute of Fundamental and Interdisciplinary Sciences, Institute of Mathematics and Physics, Beijing Union University, Beijing 100101, China; ldthuijuan@buu.edu.cn; 4 School of Chemistry and Life Sciences, Suzhou University of Science and Technology, Suzhou 215009, China; zhaoxin_sz@usts.edu.cn; 5 Basic Department, Jiyuan Vocational and Technical College, Jiyuan 454682, China; humengjia@126.com (X.L.); 1000263@jyvtc.edu.cn (W.H.)

**Keywords:** surface-enhanced Raman scattering, lyotropic liquid crystal, duty cycle, silver nanoflower

## Abstract

Surface-enhanced Raman scattering (SERS) has been widely reported to improve the sensitivity of Raman spectra. Ordinarily, the laser is focused on the sample to measure the Raman spectrum. The size of the focused light spot is comparable with that of micro-nano structures, and the number of micro-nano structures contained in the light spot area (defined as duty cycle) will severely affect the spectrum intensity. In this study, flower-like silver nanostructures were fabricated with a soft lyotropic liquid crystal template in order to investigate the effect of duty cycle. They were observed under a scanning electron microscope, and their spectrum enhancement factor was computed with the obtained Raman spectrum. Then, their duty cycles were measured using a SERS substrate at different locations. A formula was derived to represent the relation between the duty cycle of the nanoflowers and the Raman spectral intensity. This work could promote the actual applications of SERS in high-sensitivity spectrum testing.

## 1. Introduction

Raman spectra have been widely used in many fields, such as analytical sciences [1,2,3,4], surface sciences [5,6,7], and biological sciences [8,9,10,11,12]. Surface-enhanced Raman scattering (SERS) has been widely explored because it can be used to improve the sensitivity of Raman spectra [13,14,15]. Different nanostructures, such as nanowires [16,17,18], nanorods [19,20,21], nanospheres [22,23], and nanoflowers [24,25,26], are fabricated to obtain a high enhancement effect of the Raman spectra. Some SERS materials are prepared with chemical methods, such as the seed-mediated-growth method [19,27,28], the wet-chemical method [29], and the hydrothermal method [30]. Although these SERS materials can be obtained easily and have low costs, their spectral enhancement effects are unstable, and repeatability is low because they are randomly distributed and even piled up [31]. SERS materials may also be fabricated through lithography, etching, and other techniques, and their nanostructures have a periodic arrangement [32,33,34]. These materials have a stable signal enhancement and a high spectrum repeatability compared with those of the randomly distributed SERS materials, but they are costly and have a complicated fabrication process.

The key problem is the fabrication of SERS materials with high stability, high repeatability, low costs, and simple processes. To solve this problem, in 2017, our group proposed a growth method assisted by a soft lyotropic liquid crystal (LC) template and successfully prepared well-distributed flower-like silver nanostructures with high sensitivity [35,36]. In 2019, Li et al. fabricated low-cost and recyclable 3D shell-core nanostructures through a chemical reduction reaction [37]. These low-cost and simple preparation methods further promote the applications of SERS materials. In this study, a silver SERS substrate is prepared through a growth method assisted by a soft lyotropic LC template.

**Figure 1:**
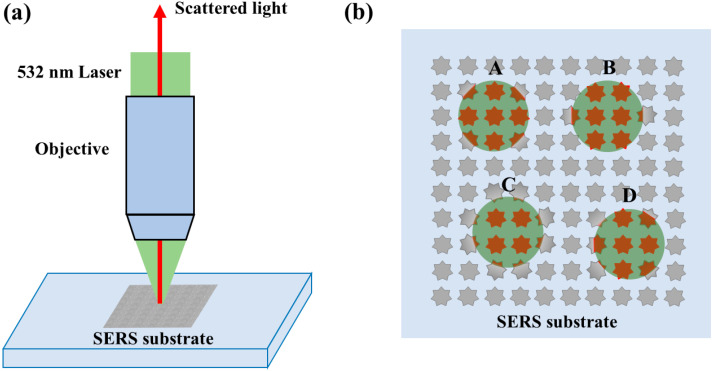
Diagram of a Raman spectrometer: (**a**) Optical setup; (**b**) Different laser focused positions on the SERS substrate.

A laser collimator lens group is typically used to emit exciting light and collect Raman scattering light in a Raman spectrometer (Figure 1a). The laser beam is focused on the surface of the SERS substrate by using the lens. As such, a valid exciting area is about tens of square microns, which is comparable with the size of a nanostructure. Regardless of the distribution of SERS substrates, some places have no nanostructure materials, as shown in Figure 1b. While the light is focused on different areas of the SERS substrate such as A, B, C and D, the different numbers of nanoparticles are contained within the illuminated area and the definition of the duty cycle of nanostructures is used to describe this situation. The enhanced effect is related to the nanostructures [38,39,40], so different enhancement effects are obtained while the exciting light is irradiated at different places. Hence, we should consider the effect of the duty cycle of nanostructures on the spectral enhancement effect.

In this study, the surface enhancement effect of a SERS substrate with different duty cycles of a nanostructure material was investigated. A flower-like silver nanostructure substrate was first fabricated and observed under a scanning electron microscope (SEM). Then, the enhancement factor (EF) was measured through Raman spectroscopy. The duty cycle of the nanomaterials was measured, and the relation between duty cycle and spectral intensity was described by obtaining its corresponding formula.

## 2. Materials and Methods

### 2.1. Materials

The following materials were used in this study: anionic surfactant sodium bis (2-ethylhexyl) sulfosuccinate (AOT; 98 wt%), oil-phase *p*-xylene (99 wt%), and AgNO_3_ (99 wt%; Sigma-Aldrich, St. Louis, MO, USA); silver foil (2.0 mm, 99%; Alfa Aesar, Alpha New Jersey, NJ, USA); rhodamine 6G (R6G; Aladdin Company, Shanghai, China); and indium tin oxide (ITO) glass (Gulo Glass Company, Luoyang, China). Deionized water and analytical-reagent ethanol were used in the experiment to clean the SERS substrate.

### 2.2. Fabrication of Flower-like Silver Nanostructures

**Figure 2:**
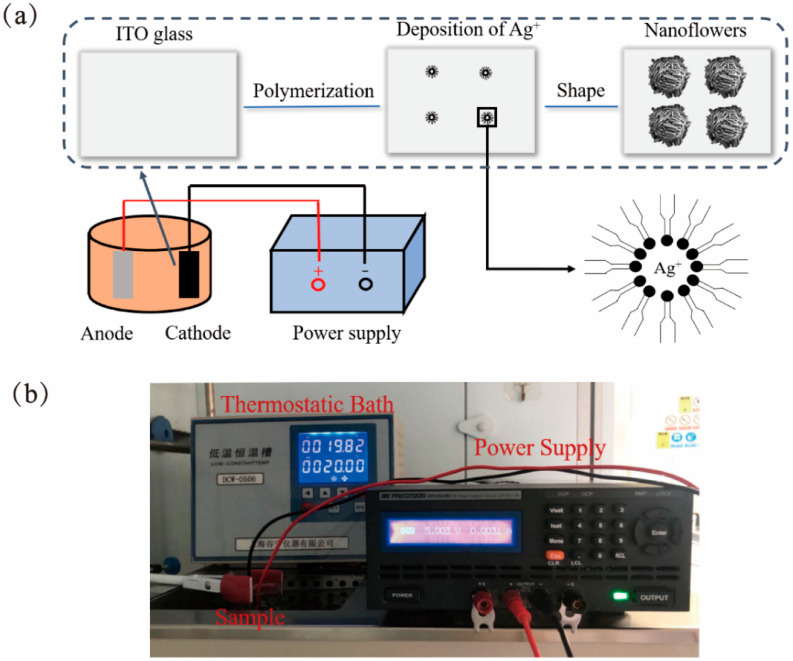
(**a**) Growth of flower-like silver nanostructures and (**b**) experimental equipment for electrodeposition.

Lyotropic LC containing the surfactant (AOT), oil-phase xylene, and water were used as a soft template to fabricate flower-like silver nanostructures. Its water content was replaced with a silver nitrate aqueous solution. Electrodeposition was performed in order to grow silver flower-like nanomaterials, and Ag^+^ was surrounded by lyotropic LC (Figure 2). The soft LC template constrained the flower-like silver nanostructures so that they could grow locally and orderly.

First, lyotropic LC was compounded with an aqueous solution of AOT, *p*-xylene, and AgNO_3_. AOT was dissolved in *p*-xylene with a 1.4 M concentration by stirring it continuously. Then, a 0.4 M AgNO_3_ aqueous solution was added dropwise to the mixture of AOT and *p*-xylene and stirred for 2 h until the mixture became a clear reddish-brown liquid. In accordance with our previous work, the cubic-phase state of the lyotropic LC was obtained in order to prepare a silver SERS substrate [35] by allowing the mixture to stand for 2 h. A silver plate and an ITO glass were used as the anode and the cathode, respectively, in the electrodeposition. The gap between the silver plate and the ITO glass was controlled at 0.7 mm by the pasting of a plastic sheet. A low-temperature thermostat bath was utilized in order to control the working temperature, and the DC voltage was supplied by a DC-stabilized power supply. Figure 2a shows the growth of flower-like silver nanostructures. Initially, Ag^+^ bound in lyotropic LC was gradually nucleated during the electrodeposition. After 3 h of electrolysis, the Ag^+^ eventually broke through the template and grew into silver nanoflowers on the cathode. Figure 2b presents the experimental equipment for the electrodeposition. The ambient temperature was controlled at 20 °C, and 5 V of DC voltage was applied between the anode and the cathode. The ITO glass with flower-like silver nanostructures was separated from the silver plate, cleaned with absolute ethanol for 15 min, and dried with a gentle flow of N_2_.

### 2.3. Characterization

The microscopic morphology of the nanostructures was characterized through thermal field emission SEM (Zeiss, Baden-Wurttemberg, Germany). The extra high tension was set to 2 kV, and the working distance was set to 4.1 mm in order to observe the nanostructures with SEM. Raman spectra were obtained using a Raman spectrometer (Horiba LabRAM, Paris, France). The working wavelength of the Raman spectrometer was 532 nm, the selected integration time was 10 s and the laser power was 0.5 mW.

### 2.4. SERS Enhancement Factor Calculation

The enhancement effect of the SERS material may be evaluated in terms of EF, which can be calculated as follows [41,42,43]:

(1) $$EF=\frac{I_{SERS}}{I_{bulk}}\times \frac{N_{bulk}}{N_{SERS}}$$

where *I_SERS_* is the intensity of the vibrational band of probe molecules on the SERS substrate, *I_bulk_* is the intensity of the same vibrational band of probe molecules on the silicon wafer, and *N_bulk_* is the average number of probe molecules absorbed on a silicon wafer. For the probe molecule solution, *N_bulk_* may be determined as

(2) $$N_{bulk}=\frac{A_{laser}VcN_{A}}{S}$$

where *A_laser_* is the area of the focused laser spot, *V* is the volume of the probe solution dripped on the silicon wafer, *c* is the concentration of the solution, *N_A_* is Avogadro’s constant, *S* is the area where the probe solution drops on the substrate, and *N_SERS_* is the average number of probe molecules absorbed on the SERS substrate. *N_SERS_* can be calculated as

(3) $$N_{SERS}=\frac{A_{laser}N_{d}A_{N}}{\delta }$$

where *N_d_* is the number density of flower-like nanostructures on the substrate, *A_N_* is the surface area of one nanoflower, and δ is the area occupied by a single probe molecule adsorbed on the substrate. When Equations (2) and (3) are substituted into Equation (1), *EF* may be rewritten as

(4) $$EF=\frac{I_{SERS}}{I_{bulk}}\times \frac{Vc\delta N_{A}}{N_{d}A_{N}S}$$

In this work, R6G was used as a Raman probe to detect Raman spectroscopy. An R6G solution was dropped onto the silicon wafer (10 mM) and SERS substrate (1 μM). After the R6G solution was completely dry, the silicon wafer and SERS substrates were used as samples for Raman spectroscopy.

### 2.5. Test and Computation of the Duty Cycle

Even if the SERS substrate has the same distribution, the number of nanoparticles within the illuminated area varies when the exciting light illuminates it at different locations, because the size of the exciting light area is comparable with that of the nanostructures. As a result, different enhancement effects of the Raman spectra occur. A concept called the duty cycle of nanostructures is defined in order to describe the number of nanoparticles within the illuminated area quantitively. For calculation simplicity, if the SERS substrate is a two-dimensional planar structure, the duty cycle of nanostructures is defined as

(5) $$D_{nano}=\frac{S_{nano}}{S_{exciting}}$$

where *S_nano_* and *S_exciting_* are the areas of the nanostructure and laser spot, respectively. Therefore, they should be obtained in order to calculate the duty cycle of nanostructures.

**Figure 3:**
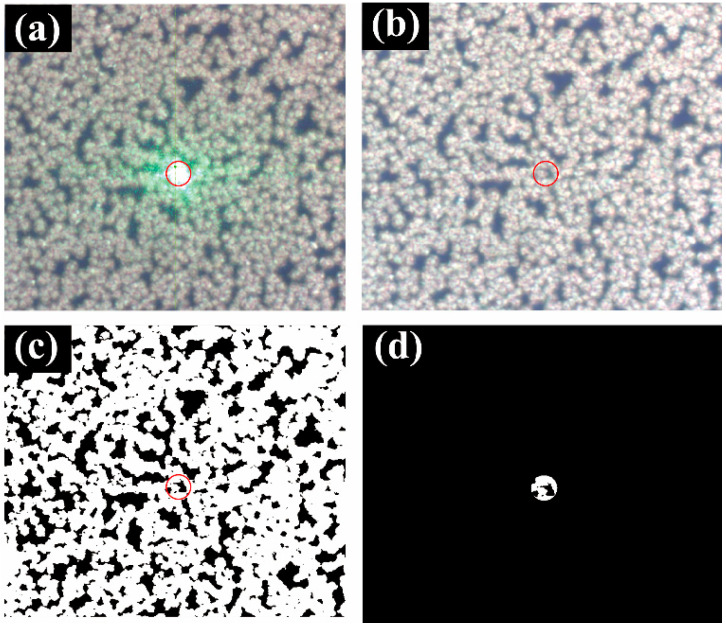
Pictures for duty cycle calculation: (**a**) Laser is turned on and (**b**) off; (**c**) Binarized image; (**d**) Testing area.

**Figure 4:**
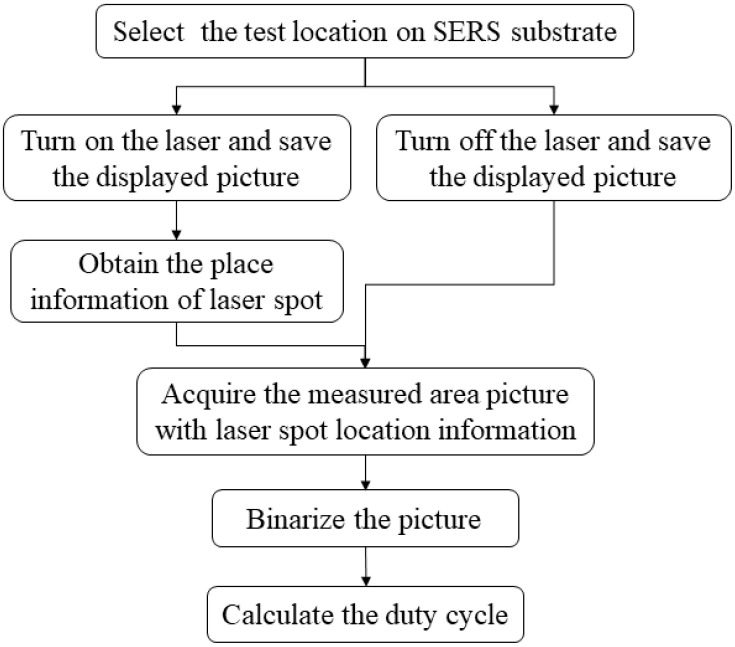
Calculation flow chart of duty cycles.

The test optical setup of the Raman spectrometer was similar to that shown in Figure 1. First, the illumination position and area of the exciting beam were ensured in order to compute the duty cycle. When the laser was turned on, the picture that is illustrated in Figure 3a was obtained using the control computer of the Raman spectrometer. From this image, the position and size of the laser spot were determined. *S_nano_* was calculated by turning off the laser and then imaging the SERS substrate at the same place. As shown in Figure 3b, the nanostructures were represented by bright sections that may be extracted with image processing. Then, the image was converted into grayscale in order to distinguish between the flower-like silver nanostructures and the gaps between structures. The grayscale image was further converted into a black and white image through binarization in order to obtain more accurate information, as shown in Figure 3c. Moreover, the position of a laser spot was used as the mask, and only the data in the mask were retained, as shown in Figure 3d. White areas denote the flower-like silver nanostructures, and black areas correspond to the gaps between nanostructures. From this picture, duty cycles were calculated, and the whole calculation flow chart is shown in Figure 4.

To obtain the universal rule of the effect of duty cycle, different laser spot sizes were used to do the experiment. This was realized by changing the objective lens of the Raman spectrometer. The 20×, 10× and 5× objective lenses were selected and the corresponding laser spot sizes were 10, 20 and 40 µm respectively.

## 3. Results and Discussions

### 3.1. Characterization of Flower-like Silver Nanostructures

**Figure 5:**
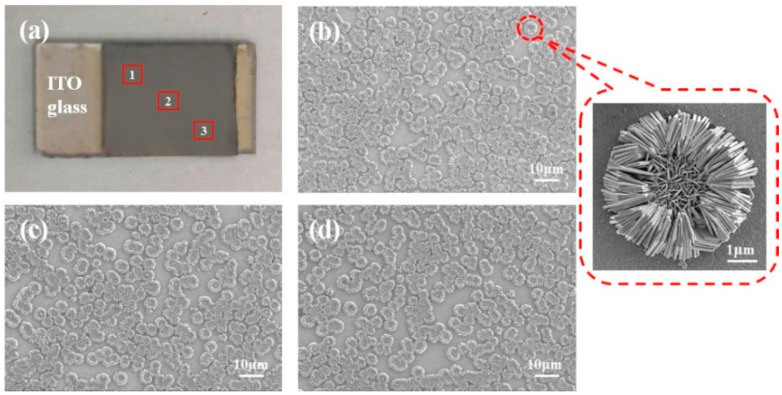
(**a**) Fabricated SERS substrate. (**b**–**d**) SEM images of the silver flower-like nanostructures in three different positions (scale bar 10 μm).

The silver flower-like SERS substrate was prepared using the growth method assisted by the soft lyotropic LC template, and its SEM images are shown in Figure 5. The gray part of the ITO glass is the prepared flower-like silver nanostructure (Figure 5a), and the SEM images of three positions are presented in Figure 5b–d. These results reveal that the flower-like silver nanostructures grew on the ITO glass substrate with uniform distribution. A magnified image of a single silver nanoflower with a measuring scale of 1 μm is illustrated in Figure 5. The diameter of the silver nanoflower is about 4 μm, and the petals with a thickness of approximately 100 nm are arranged. The silver nanoflower has many sharp inflection points, so the spectral signal enhances greatly because of the hotspot effect.

### 3.2. Enhancement Factor of SERS Substrate

**Figure 6:**
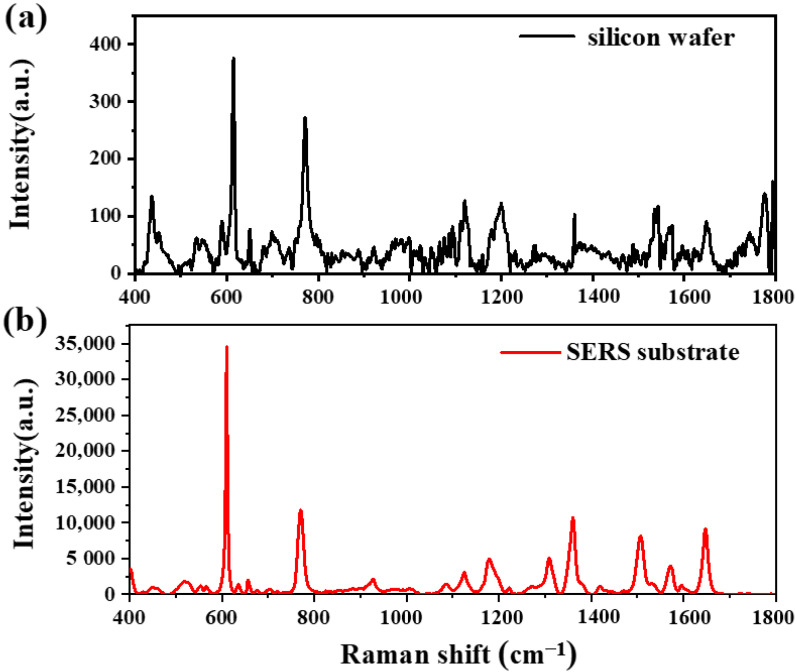
Tested Raman spectra of R6G: (**a**) Silicon wafer and (**b**) SERS substrate.

R6G is used as a Raman probe in order to obtain the Raman spectra and the measured results are shown in Figure 6. The Raman characteristic peaks of R6G are the wave numbers of 611, 774, 1183, 1312, 1361, 1511, and 1651. The spectral intensities of the silicon wafer and SERS substrate are illustrated in Figure 6a and Figure 6b, respectively. According to Equation (4), the peak intensities of the Raman spectra, namely, *I_SERS_* and *I_bulk_*, should be known to calculate the EF. The peak intensity with the wave number of 611 cm^−1^ is selected for the computation, and *I_SERS_* and *I_bulk_* are 34,575 and 374, respectively. The volume of the R6G solution (1 μM) is 3 μL. The area *S* occupied by the R6G solution on the substrate may be acquired on the basis of the area’s diameter of 2.8 mm. The value of δ is 1.88 nm^2^, and *N_d_* × *A_N_* is 0.7. Thus, the possible EF of the prepared SERS substrate is 7.3 × 10^5^, which indicates that the substrate has a strong enhancement effect. In the following figure, all of the EFs are measured and computed with this method.

### 3.3. Effect of Duty Cycle on Spectral Intensity

**Figure 7:**
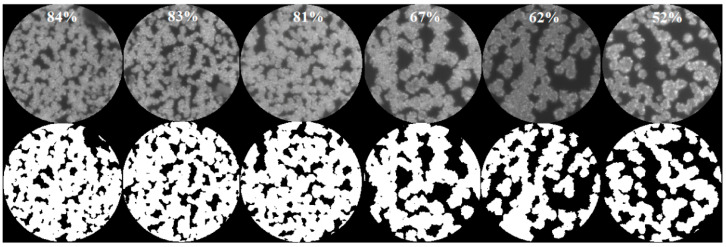
Measured and binarized images of substrate A for duty cycle calculation with 40 μm laser spots.

A silver flower-like SERS substrate (labelled with substrate A) was fabricated for the duty cycle test, and different laser irradiation areas were obtained by moving the substrate to different places. Furthermore, the laser spot sizes of 10, 20 and 40 μm were chosen to test the Raman spectrum. First, the duty cycle of the nanostructures was examined and computed for 10, 20 and 40 μm laser coverage areas. As an example, the results are shown in Figure 7 with the laser spot of 40 μm. The upper and lower parts of each subfigure are the grayscale and binarized images, respectively.

**Figure 8:**
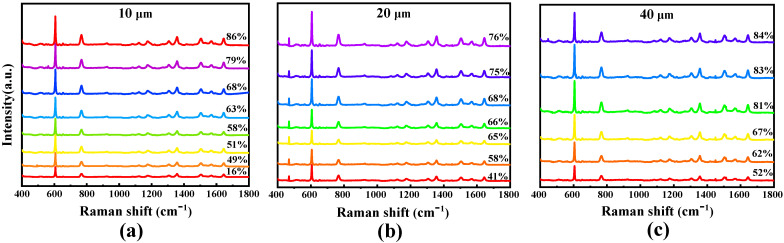
R6G Raman spectra of substrate A with different duty cycles: (**a**–**c**) 10, 20 and 40 μm spot sizes.

**Figure 9:**
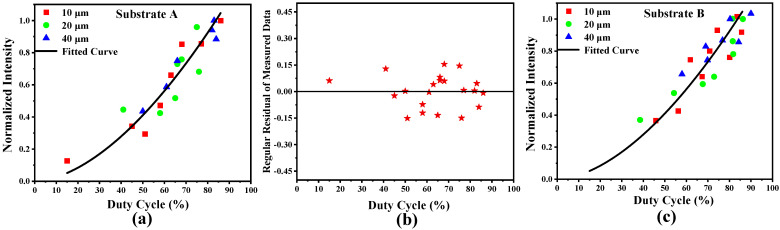
Normalized spectral intensity (**a**) and regular residual (**b**) of substrate A as functions of duty cycle, and the normalized spectral intensity of substrate B (**c**): ■, ● and ▲ represent the measured discrete data for 10, 20 and 40 μm laser spots, respectively; the real line denotes the fitted curve; the residual data is represented by ★.

R6G was also used as the probe, and the tested Raman spectra of substrate A are shown in Figure 8, which correspond to the laser spots of 10, 20 and 40 μm, respectively. This result indicates that the peak spectral intensities strengthen while the values of duty cycles increase. The peak intensity at the wave number of 611 was selected for data analysis and fitting in order to investigate the relation between the spectral intensity and the duty cycle. The measured spectral intensity varied with different laser spot sizes, so the spectral intensity was normalized for convenient comparison. The normalized spectral intensity as a function of duty cycle is presented in Figure 9a, where ■, ● and ▲ represent the measured discrete data for 10, 20 and 40 μm laser spots, respectively, and the real line is the fitted curve. For different laser spot sizes, the trend of changes in the fitted curve is similar. The fitted Raman spectral intensity may be expressed as

(6) $$I=1.3\;\;r^{1.6}$$

where *r* is the duty cycle of nanostructures. By using Equation (6), the normalized spectral intensity may be computed easily with different duty cycles. After fitting, the deviation between the measured and the fitted data was calculated as shown in Figure 9b. It indicates that the deviation magnitude belongs to [−15,15]. Further, the regular residual data were analyzed and their mean value and standard deviation are 0.002 and 0.09, respectively. In addition, some other SERS substrates were measured and the fitted results had the similar law. As an example, the measured data and fitting curve of another substrate (substrate B) are shown in Figure 9c. Similarly, the deviation magnitude is limited in [−14,15] and the standard deviation is also 0.09. It illustrates that, with the proposed method, the measuring error of the Raman spectrum intensity may be less than 10%. Hence, combining the measured data and Formula (6), the spectrum intensity may be acquired and the effect of duty cycle will be reduced greatly.

**Figure 10:**
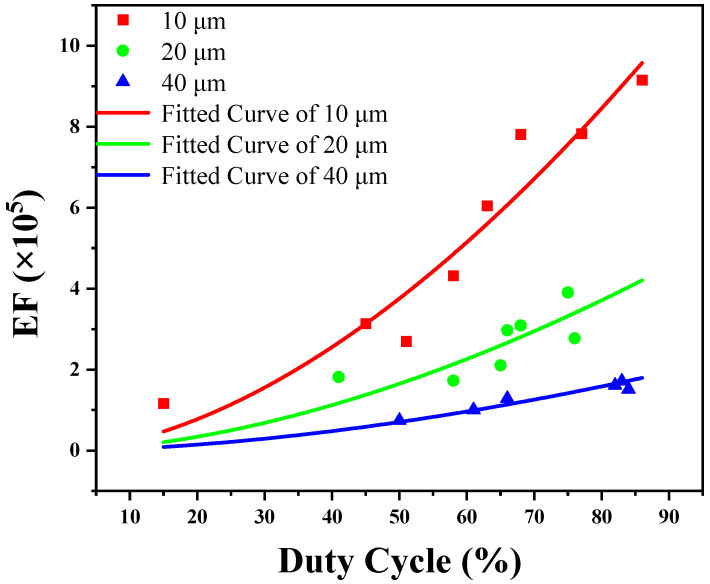
Calculated enhancement factor of substrate A: ■, ● and ▲ represent the measured discrete data for 10, 20 and 40 μm laser spots, respectively; the real line denotes the fitted curve.

The effect of duty cycle on the EF may be calculated with Equation (4), and the results of substrate A are shown in Figure 10. In contrast to the normalized spectral intensity, different laser spot sizes have various fitted EF curves. Moreover, the EF under the 10 μm spot is greater than that under the 20 and 40 μm spot because the larger the light spot is, the weaker the spectral intensity will be. For practical applications such as trace element tests, spectral intensities must be considered because they represent the number of elements. Therefore, Equation (6) may be used to measure the amount of matter for many kinds of test applications. The EF has no effect on test applications because it is only used to evaluate the enhancement ability of the SERS substrate.

## 4. Conclusions

The effect of the duty cycle of nanostructures on the Raman spectral intensity of SERS was studied. First, flower-like silver nanostructures were fabricated with a soft lyotropic LC template. SEM images show that their diameter was about 4 μm, and the thickness of their petals was approximately 100 nm. R6G was used to measure the enhancement effect of the SERS substrate, and the EF was up to 7.3 × 10^5^.

Then, multiple pictures measured with the Raman spectrometer were selected and processed to calculate the duty cycle of the nanostructures. The corresponding Raman spectrum was also tested. For laser spots of 10, 20 and 40 μm with different SERS substrates, the relation between the duty cycle and spectral intensity was obtained with the same fitting curve and described by using the established formula. The deviation magnitude between the measured and fitted data belongs to [−15, 15] and the standard deviation of regular residual data is 0.09. The measuring error of the Raman spectrum intensity is less than 10%.

This formula may be used to calibrate test sensitivity, and the SERS substrate is utilized to measure the amount of matter in many kinds of test applications. Moreover, these flower-like silver nanostructures may be easily prepared with lyotropic LC and have a low cost, and they may be used as SERS substrates for Raman spectrometers employed in various test applications. This work may serve as a reference for the quantitative calibration of SERS substrate sensitivity and promote the actual applications of SERS in high-sensitivity testing.

## Data Availability

Not applicable.

## Sample Availability

Samples of the compounds are available from the authors.
